# Future-use consent and human virome research: ethical challenges

**DOI:** 10.1128/jvi.00420-26

**Published:** 2026-07-20

**Authors:** Inmaculada de Melo-Martín, Hetanshi Naik, Joseph Yracheta, Jennifer Li-Pook-Than

**Affiliations:** 1 Division of Medical Ethics, Weill Cornell Medicine–Cornell University 12295, New York, New York, USA; 2 Department of Genetics, Stanford University, School of Medicine 480857, Stanford, California, USA; 3 Native Biodata Consortium, Eagle Butte, South Dakota, USA; Universiteit Gent, Merelbeke, Belgium

**Keywords:** ethics, informed consent, virome research

## Abstract

Future-use consent frameworks enable broad research on biospecimens and now extend to human virome studies. However, virome research challenges the core assumptions underlying these consent models. We examine four dimensions of concern: evolving interpretive frameworks for biospecimens, the unforeseen sensitivity of viral findings, unanticipated downstream uses of samples, and unresolved questions regarding the return of results. These features call for updated governance capable of sustaining the ethical legitimacy of clinical research in rapidly evolving scientific landscapes.

## INTRODUCTION

Study consent forms often include provisions allowing future unspecified research use of biospecimens. These provisions have enabled the large-scale collection and secondary analysis of human biological materials across various “omic” domains. Advances in sequencing and computational tools have expanded this model to human virome research, which has enabled the characterization of viral diversity across environmental and host-associated samples. This has contributed to knowledge about basic virology, clinical diagnostics, and public health surveillance efforts (1, 2).

These consent frameworks, however, were developed in the context of genomic and multi-omic research that treated biospecimens as sources of stable, individual biological traits, and therefore as posing relatively limited risks to participants (3). Subsequent developments in genomics have exposed key limitations of this model. For instance, genomic work has revealed greater identifiability, familial and group implications, and the analytic power to generate unanticipated inferences, undermining the assumption that future-use consent could reliably anticipate downstream harms (4, 5). But research on the human virome exposes and amplifies ethical and governance gaps in future-consent frameworks that go beyond those already identified in genomic and microbiome research. Virome data encompass pathogenic, commensal, bacteriophage, endogenous, oncogenic, and zoonotic viruses that reflect dynamic biological, social, and ecological relationships (6, 7). Viral findings can carry clinical, social, and population-level significance (8) (Table 1).

**TABLE 1:** Virus types and contextual implications

Virus type	Example	Associated implication
Pathogenic viruses	Chicken pox (varicella-zoster); SARS-CoV-2	Infection (9, 10)
Commensal or co-evolved viruses	Anelloviruses; adeno-associated virus	Regulate microbial communities (6)
Human endogenous viruses (HERV) and non-retroviral endogenous elements (NERVE)	Human endogenous retrovirus K; bornavirus-like elements; adeno-associated virus	Ancient viral elements integrated into the human genome (6); majority considered “benign,” may relate to immunity (11)
Oncoviruses and immunomodulatory viruses	Epstein–Barr virus; human papillomavirus; influenza	Linked to cancer and immune dysregulation (12)
Environmental or zoonotic viruses	SARS-CoV-2; Nipah; Hanta	Cross-species transmission (10, 13)
Viral elements integrated in the human genome (somatic or germline)	Hepatitis B virus (somatic integration); adeno-associated virus (rare integration)	May disrupt host genes, alter regulation, or contribute to oncogenesis (14)
Bacteriophages and mycoviruses	crAssphage; TmPV1	Infect bacteria; influence human microbiome and serve as reservoirs for horizontal gene exchange (15)

Although samples biobanked under future-use consent provide invaluable opportunities for virome research, we argue here that, because of the mismatch between institutional practice and participant expectations, their use exposes tensions in existing consent frameworks. We examine four dimensions of virome research with such materials that test the limits of what these future-use consent can ethically authorize: evolving interpretive frameworks for biospecimens, the unforeseen sensitivity of viral findings, unanticipated downstream uses of samples and data, and unresolved questions regarding the return of results. These features reveal structural limitations in future-use consent models and call attention to the need for adaptive oversight strategies within existing institutional frameworks that are capable of sustaining ethical research and nurturing public trust.

## FUTURE-USE CONSENT

Consent forms now routinely permit future, unspecified use of data and biospecimens, reflecting the impracticality of recontacting participants in large, longitudinal studies (3). Growing expectations for data and specimen sharing have further incentivized open-ended, prospective permission. Oversight of research conducted with these materials is entrusted to researchers, institutional review boards, and governance committees, who are expected to ensure that the research aligns with the ethical scope of the original consent (16). In practice, many future-use consents are drafted so broadly that they authorize wide-ranging sharing with minimal substantive limits, leaving oversight bodies with little basis for meaningful review.

The ethical viability of future-use consent models depends fundamentally on trust: that participants’ contributions will be used responsibly for the public good and that institutional safeguards will protect participants from harm. It also rests on two key assumptions: that participants can form a reasonable understanding of the types of research their samples might support, even if specifics are unknown; and that the nature of future research is generally predictable and falls within what participants might reasonably foresee when consenting (3).

Although these assumptions provide the ethical foundation for future-use consent, a growing body of empirical research suggests they are not consistently met in practice. Participants frequently misunderstand the scope of “future research,” overestimate the degree of control they retain over downstream uses, and are often unaware of how samples and data may circulate across institutions or be used in contexts far removed from the original study (17, 18). These limitations appear even in well-established biobank and genomic settings where broad consent is now routine.

While such shortcomings already strain traditional genomic and electronic health record research, we argue here that virome research with biobanked materials exposes deeper challenges. Viral analyses may generate findings about infection, exposure, transmission networks, or ecological circulation that introduce forms of interpretation, sensitivity, and data sharing not contemplated when initial consent was obtained. The gap between what participants reasonably understand at enrollment and what virome analyses can reveal is, thus, likely to be wider than in other omics domains.

## VIROME RESEARCH AND THE LIMITS OF FUTURE-USE CONSENT

To understand how the use of these samples raises ethical challenges that extend well beyond the lack of virome-specific language in consent documents, we must examine whether such use undermines the assumptions that give future-use consent its ethical force (3, 19). Broad consent provisions rely on shared expectations among participants, investigators, and oversight bodies about how biospecimens will be interpreted, what information they will generate, the institutional contexts in which they will circulate, and whether or how findings will be returned (Table 2). In the sections below, we argue that virome research using samples collected under future-use consent challenges each of these expectations.

**TABLE 2:** Ethical challenges in virome research and responses

Ethical challenge	Description/example	What it challenges	Proposed responses
Changing interpretive frames	Viral data reflect exposures, social contacts, and ecological networks Samples often focus on the virus rather than the donor	Assumption that samples reflect only individual biology Boundaries between human subject vs pathogen research	Clarify expectations for virome analyses under future-use consent Identify analyses that align with those expectations Flag relational/public-health analyses for proportionate administrative oversight
Unforeseen sensitivity	Viral findings can reveal infections, transmission pathways, or social relationships, risking stigma, discrimination, or legal harm	Expectation that research will not produce socially sensitive information Assumption that findings are mainly about individual health status	Standardize pathways for handling viral findings Adopt a virome-specific actionability framework Minimize human reads when human genomic information is unnecessary Secure NIH Certificates of Confidentiality
Unanticipated uses	Samples may be repurposed for public-health surveillance, outbreak detection, dual-use research, or population monitoring Oversight norms vary	Scope of consent and oversight Traditional understanding of health research	Define governance for downstream uses of virome data Require tailored institutional review pathways for new uses Set sharing, retention, and restriction criteria consistent with consent
Return of results ambiguity	Existing consents often omit whether/how viral findings will be returned Actionability depends on context	Norms for disclosure Researchers’ responsibilities Understanding of actionability	Implement virome-specific actionability categories (clinical, mandatory reporting, and non-actionable) Use standardized pathways for return, report, or withholding

### Changing interpretive frames for biospecimens

A central premise of future-use consent is that participants can reasonably understand, at the time of authorization, the general types of research that will be conducted with their samples (3, 20). Consent documents usually describe this research in terms of genetics, biomarkers, and in some cases microbiomes. Such descriptions implicitly frame biospecimens as sources of information about internal biological processes, that is, human DNA, host physiology, or relatively stable microbial communities. Within this frame, specimens are understood as revealing something about the individual body, even when analyzed at scale or over time. Virome research complicates this framing. First, viral sequences identified in blood, stool, or respiratory samples often originate outside the body and reflect exposure, contact, and circulation across social, institutional, ecological, and evolutionary contexts (21). Specimens collected for molecular profiling can later be used to infer exposure to circulating viruses, contact with infected individuals, or proximity to animal reservoirs. Hence, samples function less as a record of the donor’s internal biological state and more as a trace of interactions beyond the individual.

Second, virome analysis also changes the unit of interpretation. Genomic and microbiome research typically treat the individual as the primary unit of analysis, even when results are aggregated. Virome research, by contrast, frequently treats an individual sample as one node within a larger network of transmission or circulation. The meaning of a viral sequence often depends on its relationship to other sequences in a population across time and space because phylogenetic and comparative analyses are used to infer evolutionary relationships, host associations, and transmission patterns across hosts, regions, and ecological gradients (22). Samples are thus interpreted relationally rather than in isolation, and their significance emerges through comparison within dynamic networks rather than the characterization of individual variation alone. For example, researchers might collect a nasal swab for a respiratory-microbiome study. The sample might contain a viral sequence that, when phylogenetically compared with local surveillance data, clusters with sequences from a recent hospital outbreak. Such an inference implicates transmission links beyond the individual donor and depends on relational network analysis rather than isolated host variation (23).

Finally, virome research blurs the boundary between research on human subjects and research on pathogens. In many studies, the primary object of interest is not the participant but the virus itself: its diversity, evolution, or movement across hosts (24). Similar dynamics can arise in other omics research, but virome studies make this shift more explicit by centering analytical attention on entities whose ethical, clinical, and political significance extends beyond the individual donor.

### Unforeseen sensitivity of viral findings

Future-use consent assumes that future research will not produce information whose social or clinical implications exceed participants’ expectations or the context in which consent was granted. Viral findings, however, can reveal sensitive individual and social information in ways largely absent from other omics research at the time that consent was obtained. First, virome data can directly indicate infections that carry immediate social or clinical consequences (25). Detection of sexually transmitted viruses, blood-borne viruses, or viruses associated with substance use, incarceration, or immunosuppression can expose participants to stigma, discrimination, or legal and employment consequences (26). This sensitivity exists even when findings are incidental, and participants are asymptomatic.

Second, viral sequence data can support inferences that go beyond individual health status. Viral phylogenetic analysis can suggest timing of infection, likely sources of exposure, or directionality of transmission (27). These inferences, which can implicate intimate partners, family members, patients, coworkers, or animals, depending on the virus and context (28), map social and environmental connections in a way that is rarely possible with host human genomic data. The ethical significance here lies not in identifying individuals, but in revealing plausible social connections, professional relationships, or novel, population-specific virus-host associations in ways that can be consequential in small communities, healthcare settings, or occupational cohorts.

These relational implications separate viral sequencing from other omics domains that map group-level environmental risks. Environmental omics data sets can reveal a population’s passive, shared exposure to a localized external hazard, for instance, but they carry no implications regarding interpersonal behavior. By contrast, high-throughput viral sequencing does not merely indicate that a population shares a pathogen; it can actively map directional chains of transmission and behavioral connections within tight-knit groups. It thus introduces social, behavioral, and stigmatizing risks regarding personal relationships that are largely absent from passive exposure models.

### Unanticipated use of samples

Broad consent provisions typically authorize future use of samples for “biomedical” or “health-related” research, implying that studies aim to understand disease mechanisms, improve diagnosis or treatment, or advance general medical knowledge. Virome research increasingly moves beyond these domains into research and application contexts that differ in purpose, oversight, and consequences from those participants were likely to imagine. This shift creates a governance gap that must be addressed to preserve long-term public trust in biobanks. First, virome data can be used for population-level monitoring (29). Virome data sets derived from human samples are now central to outbreak detection, viral evolution tracking, and early warning efforts (30). In these settings, samples function not to understand the donor’s health, but to monitor viral dynamics at scale. Although these population surveillance methods are standard practice in dedicated epidemiological studies, their integration into general future-use biobanks creates a mismatch with the individual, medical research expectations that research participants are likely to have.

Second, downstream uses of virome data are often governed by different institutions or bodies that operate under legal and policy frameworks that differ from those governing academic research, including different rules for data retention, sharing, and response (31). A data set or specimen shared for research can be requested by public health authorities under statutory reporting rules, transferred into national surveillance programs with distinct retention and disclosure policies, or accessed by international consortia operating under separate data-sharing agreements. These shifts matter because they change who decides about reuse, what privacy protections apply, and what obligations, such as mandatory reporting or long-term retention, attach to the data. Unlike traditional academic biobanks, these external systems operate under distinct statutory obligations, where data management is dictated by public health statutes and international compliance agreements rather than standard research oversight. Participants were not asked to consider that their samples or associated data might enter institutional environments with different mandates, accountability structures, and expectations about how information will be used.

Third, virome research amplifies dual-use concerns because of the level of technical detail it produces. High-resolution viral sequence data are essential for understanding viral evolution, host adaptation, and immune escape, but they can lower barriers to reconstructing or modifying viral agents (32). Virome research is distinctive in combining discovery-driven sequencing, global data sharing, and rapid dissemination. Researchers working with biobanked samples may therefore contribute to data sets that are scientifically valuable but also usable in contexts, e.g., biodefense or security research, that fall outside what participants typically authorize when consented to “health-related” research. While biosecurity concerns exist across life sciences, participants donating samples for general research are unlikely to anticipate that their individual biospecimens could serve as foundational blueprints for tracking or synthesizing infectious pathogens.

Fourth, virome data are inherently relational: they describe the microbial and viral interactions within communities, households, animals, and ecosystems (6). These collective and ecological dimensions fall outside the individualistic assumptions that underlie most consent processes. Participants may not reasonably have anticipated that their samples could contribute to broad assessments of community-level or cross-species risk, or that their data would be used to map environmental viral circulation in ways that extend far beyond the traditional focus of consent frameworks.

### Returning virome research results

A fourth area where virome research tests the limits of future-use consent involves the return of results. Future-use consent typically takes one of two positions regarding return of results: either no individual results will be returned, or only results that are clinically actionable and relevant to the participant’s health will be disclosed (33). These policies were formulated without virome research in view, and they create distinct ethical and practical problems for researchers. First, the absence of virome-specific language produces uncertainty about whether viral findings fall within the scope of existing return-of-results policies (34). Participants were not told that their samples would be screened for viruses or that viral infections might be detected incidentally. When a consent form states that no results will be returned, it is typically understood in the context of genetic variants or biomarker data. It is questionable whether participants would have understood this language to include viral infections, particularly those that are treatable, transmissible, or well known.

Second, strict non-disclosure may conflict with widely accepted norms in virology and infectious disease research. In many areas of virology, detection of a clinically relevant virus carries an expectation of follow-up, referral, or reporting, even outside formal diagnostic settings (35). When virome research identifies an infection that could warrant clinical follow-up, researchers may experience strong moral pressure to disclose. Although human genomic results face a similar tension regarding incidental medical findings (16), the pressure in virome research is distinct because of real-time public health imperatives to interrupt transmission chains rather than manage individual health risks. However, disclosing these findings under a consent document that explicitly stated that no results would be returned risks overriding participant expectations and institutional commitments made at enrollment.

Third, what is “actionable” is ambiguous when applied to virome findings. Many future-use consents state that results will be returned if they are analytically valid, clinically actionable, and relevant to the participant’s health. These criteria, developed primarily for genetic findings, do not translate cleanly to virome data. For viromes, actionability is context-dependent: some viral findings are treatable; others lack treatment, but awareness can prompt monitoring or research; some are legally reportable; and still others are actionable at a population level but not for the individual. Ultimately, actionability in the virome context can expand beyond individual clinical care to encompass broader public health utility, creating a definition of “action” that standard consent frameworks simply did not anticipate.

Fourth, the lack of virome-specific consent language shifts responsibility onto individual researchers and institutions without clear guidance. In the absence of explicit authorization, decisions about whether to return viral findings are often made *ad hoc*, relying on individual judgment or institutional risk tolerance. This leads to inconsistency across studies and biobanks, creates conflicting legal and regulatory liabilities, and exposes researchers to ethical criticism. Withholding results can appear callous; returning them can disrespect participants’ autonomy. The problem is not researcher indifference, but a consent framework that never anticipated virome findings.

## MOVING FORWARD

The challenges posed by virome research are especially difficult to navigate because the samples at issue were collected under consent frameworks that anticipated diverse future uses but did not envision viral analyses or the distinctive issues they raise (3, 19, 20). Even so, the fact that these challenges cannot be resolved outright does not leave researchers and institutions without options. Several feasible steps can be integrated into existing institutional workflows to help reduce uncertainty, promote consistency, and respect participants while allowing virome research to proceed responsibly (Table 2).

One useful step would be for institutions to set expectations for how virome analyses should be approached when using samples collected under future-use consent. This can include identifying categories of virome analyses that align with participants’ reasonable expectations such as analyzing how the general virome correlates with chronic health conditions. It could also involve flagging analyses that raise relational or public-health implications, such as projects mapping localized viral transmission networks, and that warrant proportionate administrative oversight. For instance, institutions could apply standard data-use agreements that restrict localized re-identification or could require the use of aggregate-reporting thresholds to protect community privacy. Institutions could also outline how researchers should handle analyses that reveal transmissible pathogens or household-level exposures (3, 17, 18). Establishing these expectations provides a practical way to ensure that virome research proceeds in a manner consistent with the spirit of broad consent while utilizing existing institutional review board (IRB) workflows rather than creating new administrative burdens.

Another useful strategy would be to establish simple, standardized pathways for handling viral results. This can include distinguishing findings that have clinical relevance, findings that trigger public-health reporting obligations, and findings that are meaningful only at a population level (16, 33, 34). Setting out these categories in advance gives researchers a clear way to decide how to manage unexpected viral signals, when some type of administrative oversight is needed, and when no action is required. A shared pathway also ensures that similar findings are handled consistently across studies, reducing *ad hoc* decision-making and mitigating variability without introducing disruptive protocol re-reviews while research is underway.

Adopting an actionability framework tailored to virome research could also be helpful (16, 33, 34). Institutions could distinguish between different types of viral information and specify what, if anything, follows from each category. For example, they can differentiate findings that have clear clinical implications, findings that trigger public-health reporting obligations, and findings that are scientifically interesting but have no individual-level relevance. Treating these categories separately allows institutions to decide when administrative clearance is needed, when reporting pathways apply, and when no further action is appropriate. An actionability framework helps ensure that virome findings are handled in a way that aligns with participants’ reasonable expectations while maintaining a predictable and transparent path forward.

Beyond managing viral findings themselves, institutions must also consider how virome data are governed once produced. One option would be to set out how virome data may be used, particularly for purposes that extend beyond the original research aims (31). This can include specifying when proposed downstream uses, such as integration into pathogen-surveillance systems, development of early-warning tools, utilization of novel immunogenetic data from isolated populations, or linkage with environmental or mobility data sets, require specialized data-use approval. Institutions can also outline criteria for when virome data may be shared with external partners, when it must remain within the research environment, and when proposed uses fall outside the reasonable expectations of consent. Establishing these parameters can help ensure that virome data are not repurposed in ways participants could not have anticipated and provides researchers with a clear process for evaluating new or evolving uses of viral information.

In addition to setting parameters for downstream uses, institutions should also adopt technical measures that reduce privacy risks inherent in virome data sets. Because virome sequencing often co-captures human DNA and RNA alongside viral material, attention to data-minimization practices is essential when working with samples collected under future-use consent (1, 6). In many virome studies, retaining human reads is unnecessary to meet the research goals, and their removal can substantially reduce the risk of generating identifiable human information. One widely used safeguard is computational “scrubbing,” in which human reads are identified and removed prior to downstream virome analysis (1, 7). Of course, some virome analyses, such as studies of host–virus interactions, viral integration, or host genetic factors that shape viral dynamics, require limited human genomic information to achieve their scientific aims (6, 11, 14). In these cases, institutions could require clear justification for retaining human reads and ensure that appropriate governance and access controls are in place (16, 31). Institutions can also secure NIH Certificates of Confidentiality (CoCs) for these data sets. CoCs do not override mandatory public health reporting, but they provide an essential legal shield against forced disclosure via third-party subpoenas, civil litigation, or law enforcement demands. Incorporating such measures into institutional guidance helps ensure that virome data sets are handled in a manner consistent with participants’ reasonable expectations and with broader commitments to privacy and responsible data stewardship.

Finally, attention to the challenges discussed here can inform the design of future sample-collection efforts for virome research. Researchers planning new studies are in a position to address these issues from the outset by explicitly including provisions for population-level surveillance, relational and behavioral tracking risks, as well as clear pathways for the return of incidental viral findings (3, 17, 19, 20). Likewise, future protocols could move beyond static consent documents and use dynamic consent models that allow participants to adjust their preferences as research goals change (36). For instance, institutions could use automated software to instantly block or allow the use of samples for new research projects based on each participant’s individual choices or provide secure online portals where individuals can log in and adjust their privacy settings in real time. Incorporating these updated consent provisions and flexible digital mechanisms into consent materials, study descriptions, and governance arrangements allows new collections to anticipate the distinctive features of virome research rather than confronting them only after analyses are underway. In this way, the difficulties encountered with legacy samples can serve as a practical guide for shaping more transparent and better-aligned practices going forward.

## CONCLUSION

Virome research exposes the limits of future-use consent models. Interpretive complexity, unforeseen sensitivity, unanticipated uses, and ambiguities around the return of results all highlight a structural mismatch between consent provisions and the realities of contemporary virome research. These gaps underscore how evolving scientific methods can transform the ethical and social significance of previously collected samples, creating challenges in ways that existing consent frameworks were not designed to accommodate. Sustaining ethical legitimacy in virome research therefore requires governance mechanisms that evolve alongside scientific advances, addressing the points where future-use consent no longer provides adequate guidance: clear actionability criteria for virome findings, tailored pathways for relational or public-health-relevant analyses, and data-sharing limits or access controls that balance openness with participant protection. Importantly, these safeguards can be operationalized through existing oversight structures, maximizing the utility of current administrative frameworks rather than introducing new regulatory burdens. Integrating these approaches alongside standard institutional stewardship, such as regular updates to consent language, oversight policies, and repository practices, ensures that institutional mechanisms remain appropriate as methods and uses change. Attending to these structural gaps is essential for ensuring that participants’ contributions are used responsibly and that trust in research is nurtured as virome science continues to expand.
